# TALE-PvuII Fusion Proteins – Novel Tools for Gene Targeting

**DOI:** 10.1371/journal.pone.0082539

**Published:** 2013-12-05

**Authors:** Mert Yanik, Jamal Alzubi, Thomas Lahaye, Toni Cathomen, Alfred Pingoud, Wolfgang Wende

**Affiliations:** 1 Institute for Biochemistry, Justus-Liebig-University Giessen, Giessen, Germany; 2 Institute for Cell and Gene Therapy, University Medical Center Freiburg, Freiburg, Germany; 3 Center for Chronic Immunodeficiency, University Medical Center Freiburg, Freiburg, Germany; 4 ZMBP – General Genetics, University of Tuebingen, Tuebingen, Germany; New England Biolabs, Inc., United States of America

## Abstract

Zinc finger nucleases (ZFNs) consist of zinc fingers as DNA-binding module and the non-specific DNA-cleavage domain of the restriction endonuclease FokI as DNA-cleavage module. This architecture is also used by TALE nucleases (TALENs), in which the DNA-binding modules of the ZFNs have been replaced by DNA-binding domains based on transcription activator like effector (TALE) proteins. Both TALENs and ZFNs are programmable nucleases which rely on the dimerization of FokI to induce double-strand DNA cleavage at the target site after recognition of the target DNA by the respective DNA-binding module. TALENs seem to have an advantage over ZFNs, as the assembly of TALE proteins is easier than that of ZFNs. Here, we present evidence that variant TALENs can be produced by replacing the catalytic domain of FokI with the restriction endonuclease PvuII. These fusion proteins recognize only the composite recognition site consisting of the target site of the TALE protein and the PvuII recognition sequence (addressed site), but not isolated TALE or PvuII recognition sites (unaddressed sites), even at high excess of protein over DNA and long incubation times. *In vitro*, their preference for an addressed over an unaddressed site is > 34,000-fold. Moreover, TALE-PvuII fusion proteins are active *in cellula* with minimal cytotoxicity.

## Introduction

Precision genome engineering requires highly specific nucleases that, even within the context of a complex genome, cleaves only a single site to prevent genotoxic off-target activity. So far, a variety of different architectures has been used to generate such highly specific nucleases [1-5]. Although some natural homing endonucleases could be engineered to fulfill this requirement [6-8], these “meganucleases“ are not easily re-programmable, in contrast to the zinc finger nucleases [9-16] introduced by Kim et al. [15]. Typically, to generate a ZFN, the non-specific DNA-cleavage domain of the Type IIS restriction endonuclease FokI is fused to an array of three or four zinc fingers, which constitute the DNA recognition module that can be varied to recognize distinct DNA sequences [10,14]. After transcription activator-like effector (TALE) proteins had been discovered [17-19] and shown that they display a simple code for DNA recognition [20-22], it became clear that the DNA binding domain of TALE proteins could be used instead of zinc finger arrays for generating customizable nucleases [23-26]. Because of their simple design, predictable sequence specificity and easy synthesis [27-30], TALE-based nucleases (TALENs) seem to become the nucleases of choice for genome engineering [31-35]. Recently, a new powerful technique for genome engineering applications has been described based on the programmable RNA-guided DNA endonuclease Cas9 from a prokaryotic type II CRISPR system [36-38]. The endonuclease is guided to the target DNA by a short RNA molecule that contains a sequence complementary to the cleavage site fused to an invariant RNA part. In contrast to ZFNs and TALENs, this architecture does not rely on the fusion with an external nuclease domain [4].

Typical ZFNs and TALENs contain the FokI DNA-cleavage module [39,40] that in its natural configuration recognizes a non-palindromic sequence and cleaves the DNA asymmetrically 9 and 13 nucleotides downstream of its recognition site [41]. Double-strand cleavage requires the dimerization of a FokI monomer bound to its recognition site via its catalytic domain with a second monomer by recruiting the latter either from solution [42] or, preferably, from another monomer bound to a second recognition site, which can be, but does not need to be, nearby [43]. In ZFNs and TALENs, the DNA-binding domains are designed to recognize two adjacent DNA sequences, and to induce cleavage of the DNA between the target sequences upon FokI dimerization. As pointed out by Halford and co-workers [44], “this strategy fails to take account of the fact that the catalytic domains of FokI can dimerize across distant sites or even at a solitary site. Additional copies of either target sequence elsewhere in the chromosome must elicit off-target cleavages”. The reaction mechanism of FokI, therefore, raises some concerns about the possibility of targeting ZFNs and TALENs to unique DNA sites. To minimize this risk, specially engineered obligate heterodimeric FokI cleavage domains have been used in ZFNs [45,46]. Indeed, a recent study had identified several off-target cleavage sites *in vivo* by a genome-wide integration site analysis that mapped the actual *in vivo* cleavage activity of four ZFN pairs targeting *CCR5* or *ILRG* genes [47]. In another study, the off-target cleavage specificities of ZFNs targeting *CCR5* and VEGF-A genes were analyzed by *in vitro* selection; several of these sites were found to be cleaved also in cultured human cells [48].

There are alternative architectures of programmable nucleases substituting the nonspecific FokI cleavage domain. We had produced a programmable nuclease by fusing a triple-helix-forming oligonucleotide (TFO) as binding module with the single-chain (sc) variant of PvuII as cleavage module and shown that this chimera had a preference for cleavage of an “addressed” site, i.e. a PvuII site flanked by a triple-helix-forming site, compared to an “unaddressed” site, i.e. a PvuII site not flanked by a triple-helix-forming site, by >1000-fold [49]. This specificity, however, was only achieved, when the substrate was pre-incubated in the absence of Mg^2+^ and the reaction started by the addition of Mg^2+^. This would exclude an application *in vivo*, unless one would “cage” the enzyme with a photo-labile group and start the reaction by “decaging” with light [50]. In addition, the TFO-scPvuII constructs can only be delivered by “profection” into cells [51,52].

The TFO-scPvuII study showed that there are alternatives to the catalytic domain of FokI as DNA-cleavage module. This was subsequently validated for protein chimeras comprising the restriction endonuclease PvuII as DNA-cleavage module in combination with an inactivated homing endonuclease, I-SceI* [53], or a zinc finger array [54] as a DNA-binding module and recently with fusions exploiting the specific monomeric nuclease TevI [55]. Using a site-specific rather than a non-specific nuclease as cleavage module in a programmable nuclease adds an extra element of specificity, given by the recognition site of the restriction endonuclease in addition to the recognition site of the DNA-binding module. This increase in specificity became evident in a comparison between zinc finger-FokI and zinc finger-PvuII fusion proteins which demonstrated that the PvuII constructs, even at high concentrations and after long incubation times, did not show off-target cleavage *in vitro* [54]. Furthermore, two recent papers showed that the nonspecific FokI cleavage domain in TALENs could be replaced by TevI, introducing double-strand breaks [56], and MutH, introducing strandspecific nicks [57].

In this study, we investigated whether variant TALENs can be produced by fusing PvuII as a DNA-cleavage module to TALE proteins. We demonstrate that with appropriate linkers PvuII-based TALENs are able to specifically cleave the addressed target sites, even at a large excess of enzyme over substrate and after long incubation times. We conclude that PvuII-based TALENs are a promising alternative to FokI-based TALENs. 

## Materials and Methods

### Design and construction of TALE-PvuII fusion proteins

The AvrBs3 TALE protein was truncated at the N-terminus by 152 amino acids and at the C-terminus by 250 and 215 amino acids, leaving 28 or 63 amino acids, respectively, after the last repeat. The truncated proteins were fused to the PvuII high fidelity variant T46G [58] or alternatively to the single-chain PvuII variant [59] with a 16 amino acid linker, L [= SSVIPNRGVTKQLVKG, based on the linker used in [54]]. All proteins contained an N-terminal Strep-tag and a C-terminal His_6_-tag. The following constructs were generated: Strep-AvrBs3-63-L-PvuII^T46G^-His_6_, Strep-AvrBs3-28-L-PvuII^T46G^-His_6_, Strep-AvrBs3-28-PvuII^T46G^-His_6_, Strep-AvrBs3-L-PvuII^T46G^-His_6_, Strep-AvrBs3-63-L-scPvuII^T46G^-His_6_, Strep-AvrBs3-28-L-scPvuII^T46G^-His_6_. 

### Protein expression and purification

For expression and purification, the *Escherichia coli* strain XL10-Gold (Stratagene) transformed with the pLGM plasmid containing the sequence for the PvuII DNA-methyltransferase was used. Cultures were induced with 1 mM isopropyl-ß-D-thiogalactopyranoside at 37°C at an OD_600nm_ of 0.7. The TALE-scPvuII^T46G^-producing cells were harvested after 4 h at 37°C, whereas the TALE-PvuII^T46G^-producing cells were harvested after an overnight induction at 23°C. The cells were resuspended in 30 mM K-phosphate, 1 M NaCl, 1 mM DTT, 1 mM EDTA, 1 mM phenyl methane sulfonyl fluoride (PMSF), 0.01% w/v Lubrol, pH 7.8 and lysed by sonification. Cell debris was removed by centrifugation (>17000 g) for 30 min at 4°C. The His_6_-tagged proteins were purified by affinity chromatography over Ni-NTA agarose (Qiagen). The Ni-NTA agarose was equilibrated with the resuspension buffer and incubated with the recombinant protein preparations for 1 h at 4°C. The first wash step was performed with the resuspension buffer and the second wash step with 30 mM K-phosphate, 500 mM NaCl, 1 mM EDTA, 1 mM DTT, 0.01% Lubrol, 15 mM imidazole and 1 mM PMSF, pH 7.8. The eluted proteins (elution buffer: 50 mM K-phosphate, 300 mM NaCl, 250 mM imidazole, pH 8) were further purified by a heparin column chromatography on an ÄKTA HPLC (GE Healthcare) using a gradient elution from 0.3 to 1 M KCl in 30 mM K-phosphate, 1 mM DTT, 1 mM EDTA, 1 mM PMSF, 0.01% Lubrol, pH 7.8. The elution of the proteins started between 500 and 550 mM KCl. Fractions with pure protein were dialyzed overnight at 4°C against 30 mM K-phosphate, 550 mM KCl, 1 mM DTT, 1 mM EDTA, 60% v/v glycerol, pH 7.8 and stored at -20°C. The protein concentration was determined by measuring the absorbance at 280 nm [the molar extinction coefficient was determined according to Pace et al. [60]]. The progress of the protein purification was monitored by sodium dodecyl sulfate-polyacrylamide gel electrophoresis analysis (Figure S1).

### Plasmid cleavage assay

To characterize the cleavage rates of the different TALE-PvuII nucleases with different targets, plasmid substrates were generated. For this purpose, the TALE-PvuII target sites were inserted as a cassette into the pAT153 vector (Table S1). The AvrBs3 recognition site had been optimized based on the RVD code according to Boch et al. [21]. As an unaddressed target, a pAT153-derived plasmid with just one PvuII site was used. For the analysis of the kinetics with near-stoichiometric concentrations of substrate and enzyme, 8 nM substrate were incubated with 8 nM active enzyme (scPvuII acts as a monomer) in 20 mM Tris-Ac, 50 or 120 mM KCl, respectively, 1 mM Mg-Ac, pH 7.5 at 37°C. Taking the enzyme dilution into account, the ionic strength of the reaction mixture was between 145-150 mM. The kinetics were analyzed after defined time points (up to 24 h) by agarose gel electrophoresis and ethidium bromide gel staining; documentation had been performed using the BioDocAnalyze system (Biometra). For competition assays, 8 nM plasmid DNA harbouring an addressed PvuII site was co-incubated with 32 nM of a 900 bp long PCR fragment harbouring an unaddressed PvuII site and with 2 nM, 8 nM, and 320 nM enzyme, respectively. Furthermore, for testing the specificity of the TALE-PvuII nuclease, we have also carried out cleavage experiments with plasmid substrates in which the AvrBs3 recognition sequence was replaced by the AvrBs4 recognition sequence which differ in 8 out of 19 positions from each other (see Table S1 for plasmid substrates used).

### Radioactively labelled PCR product cleavage assay

In order to accurately determine the preference of the TALE-PvuII constructs for the addressed site (T3-n-P-n-T3, T3-6-P, where T3 and P are the AvrBs3 target site and the PvuII site, respectively, separated by n or 6 bp) compared to the unaddressed site (-P-), radioactively labelled PCR fragments were generated using [α^32^P]dATP (Table S1). 20 nM substrate were incubated with 20 nM enzyme in 20 mM Tris-Ac, 120 mM K-Ac, 1 mM Mg-Ac, pH 7.5 at 37°C. For competition assays, 20 mM of each substrate and 20 nM enzyme were incubated. After defined time intervals, samples were analyzed by polyacrylamide gel electrophoresis. The quantification was carried out with the Instant Imager system (Packard) using the Instant Imager software. Initial rates were calculated by linear regression analysis.

### Viability assay

Electrocompetent JM109 E. coli cells, not expressing the PvuII DNA-methyltransferase, were transfected with 50 ng plasmid DNA harbouring the genes of the TALE-PvuII variants. The DNA was mixed with cells and the mixture then placed in an electroporation cuvette. The transfection was done in an electroporator (Eppendorf, electroporator 2510) at 1350 V. Immediately after transfection, cells were resuspended in 900 µl LB-medium and incubated for 1 h at 37°C. Afterwards, 50 µl of the suspension were spread on an agar plate with ampicillin and incubated overnight at 37°C. The next day, colonies were counted and normalized against the control [cells expressing TALE (AvrBs3)]. The assay was repeated with the *E. coli* strain XL10Gold (Stratagene) using 100 ng DNA for the transfection and 100 µl of the suspension per agar plate. The viability factor is defined by the ratio of the viable clones of *E. coli* cultures either expressing or not expressing the TALE-PvuII variants [61].

### Plasmid cleavage assay in HEK293T cells

HEK293T cells were cultured in Dulbecco’s modified Eagle medium supplemented with 10% fetal bovine serum and 1% penicillin/streptomycin (Invitrogen). Cells were seeded in 6-well plates at a density of 500,000 cells/well. After 24 h, cells were transfected using polyethylenimin (PEI) as described before [62] with 1 µg of addressed target (T3-6bp-PvuII-6bp-T3) and 4 µg of AvrBs3-PvuII (variants G135W, G53R, A92T), respectively. Cells were harvested 3 days after transfection, and total DNA extracted using the QIAamp DNA Mini Kit (Qiagen). The region of 517 bp surrounding the PvuII site was amplified by PCR using 50 ng of total DNA as template, along with 0.2 µM of each primer (5’-gtatcgtccattccgacagcatc and 5’-ctcgccgaaaatgacccagag), 200 mM dNTPs, and 1 U of Phusion high fidelity DNA polymerase for 30 cycles. PCR amplicons were cleaned up using the QIAquick PCR Purification Kit (Qiagen) and 100 ng DNA was subjected to digestion with 20 U of PvuII-HF (New England BioLabs). 

### Cytotoxicity assay in HEK293T cells

Nuclease-associated toxicity was determined basically as previously described [63]. Briefly, HEK293T cells were seeded in 24-well plates at a density of 120,000 cells/well. After 24 h, cells were transfected using PEI with 800 ng of each TALEN-PvuII expression vectors (AvrBs3-PvuII variants G135W, G53R, A92T) together with 100 ng of the mCherry expression vector (kindly provided by Roger Y. Tsien, UC San Diego), and 1.25 µg pUC118. As positive control, 800 ng of expression vectors encoding the toxic ZFN pair GZF1N/GZF3N [64] were applied. The cell survival rate was calculated as the decrease in the number of mCherry-positive cells determined by flow cytometry (FACSCalibur; BD Biosciences) from days 2 to 5, normalized to cells transfected with an I-SceI expression vector.

## Results

**Figure 1:**
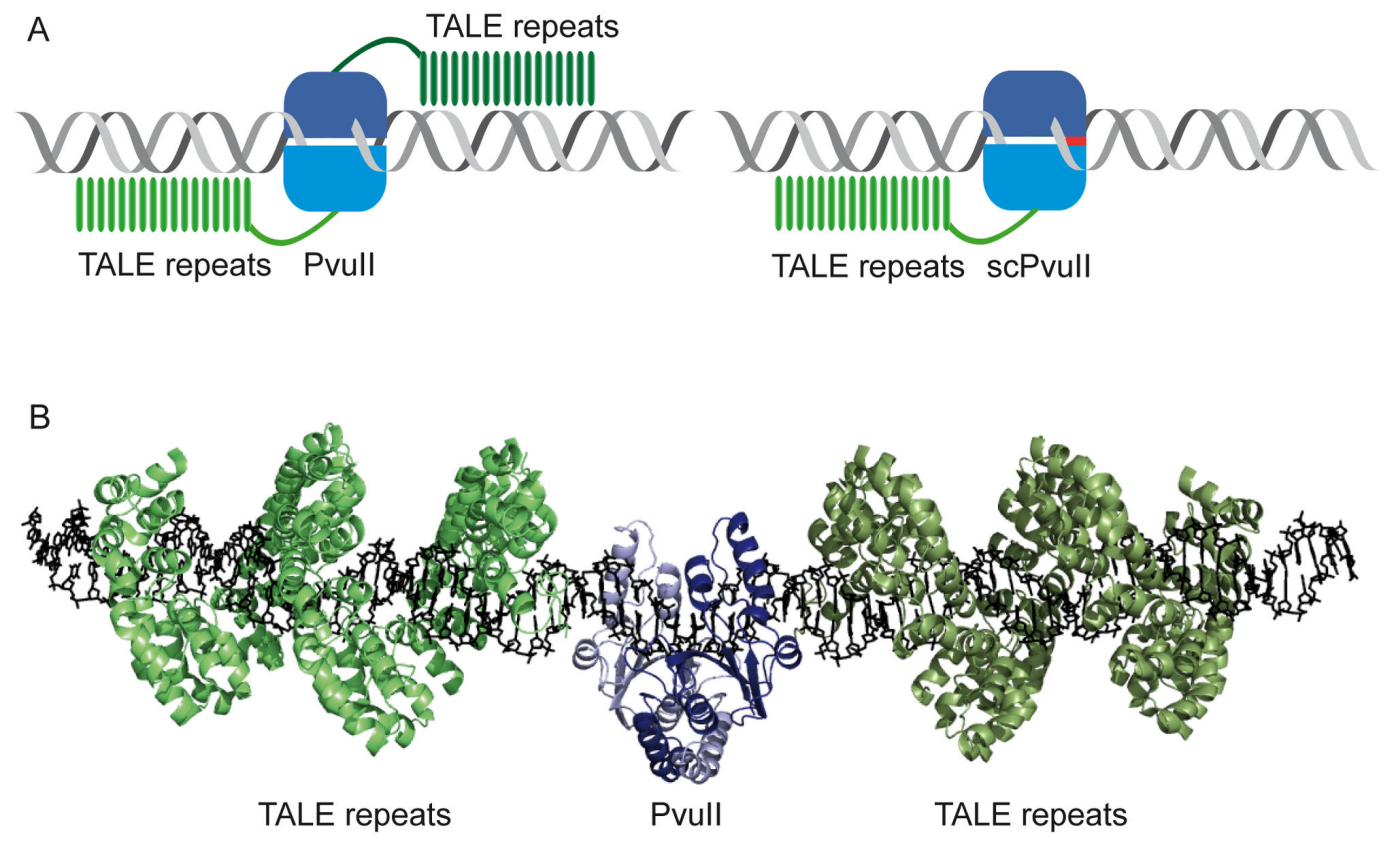
TALE-PvuII fusion proteins. (**A**) Scheme of the architecture of TALE–PvuII fusion proteins. Left: wtPvuII, a homodimer in which the DNA-binding module of a TALE protein is fused via a linker of defined length. Right: scPvuII, a monomeric nuclease in which the DNA-binding module of a TALE protein is fused via a linker of defined length. (**B**) Model of a TALE–wtPvuII fusion protein. The fusion protein is a dimer of identical subunits, each composed of a PvuII subunit and a TALE protein. This model was constructed by aligning the structures of the individual proteins [pdb 1pvi [74] and pdb 3ugm [76]] on a DNA composed of the PvuII recognition site and two TALE target sites up- and downstream of the PvuII recognition site, separated by 6 bp. The C-termini of the PvuII subunits and the N-termini of the TALE protein are separated by about 3 nm. This distance must be covered by a peptide linker of suitable length. The image was generated with PyMol.

The goal of our study was to generate a TALE-PvuII fusion protein that cleaves DNA only at a composite site consisting of the TALE target site and the PvuII recognition site (addressed site) but not at any other PvuII site (unaddressed site). For this purpose, trimmed versions of AvrBs3 [25], a well characterized natural TALE protein [18,65,66], were fused to either the homodimeric wild type PvuII or the single chain (sc) variant of PvuII, in which the two subunits of wtPvuII are covalently linked via a short peptide linker [59], as shown schematically in Figure 1A. A model of the TALE-PvuII fusion protein in complex with DNA is shown in Figure 1B.

Altogether, six different fusion proteins with PvuII and scPvuII, which all carry the T46G substitution that reduces star activity [58], were prepared (see Materials and Methods).They differed in the distance between the DNA-binding (AvrBs3 or AvrBs4) and the DNA-cleavage module (scPvuII^T46G^ or PvuII^T46G^ and variants thereof). The fusion proteins were expressed in *E. coli*, purified to homogeneity and tested for their DNA-cleavage activity towards both addressed and unaddressed PvuII sites. The ratio between these two activities is reflecting the specificity of the fusion protein. Two different types of substrates were used, plasmids and PCR products, the latter also radioactively labelled to quantify target cleavage specificity.

### Linker length determines specificity of TALE-PvuII fusion proteins

**Figure 2:**
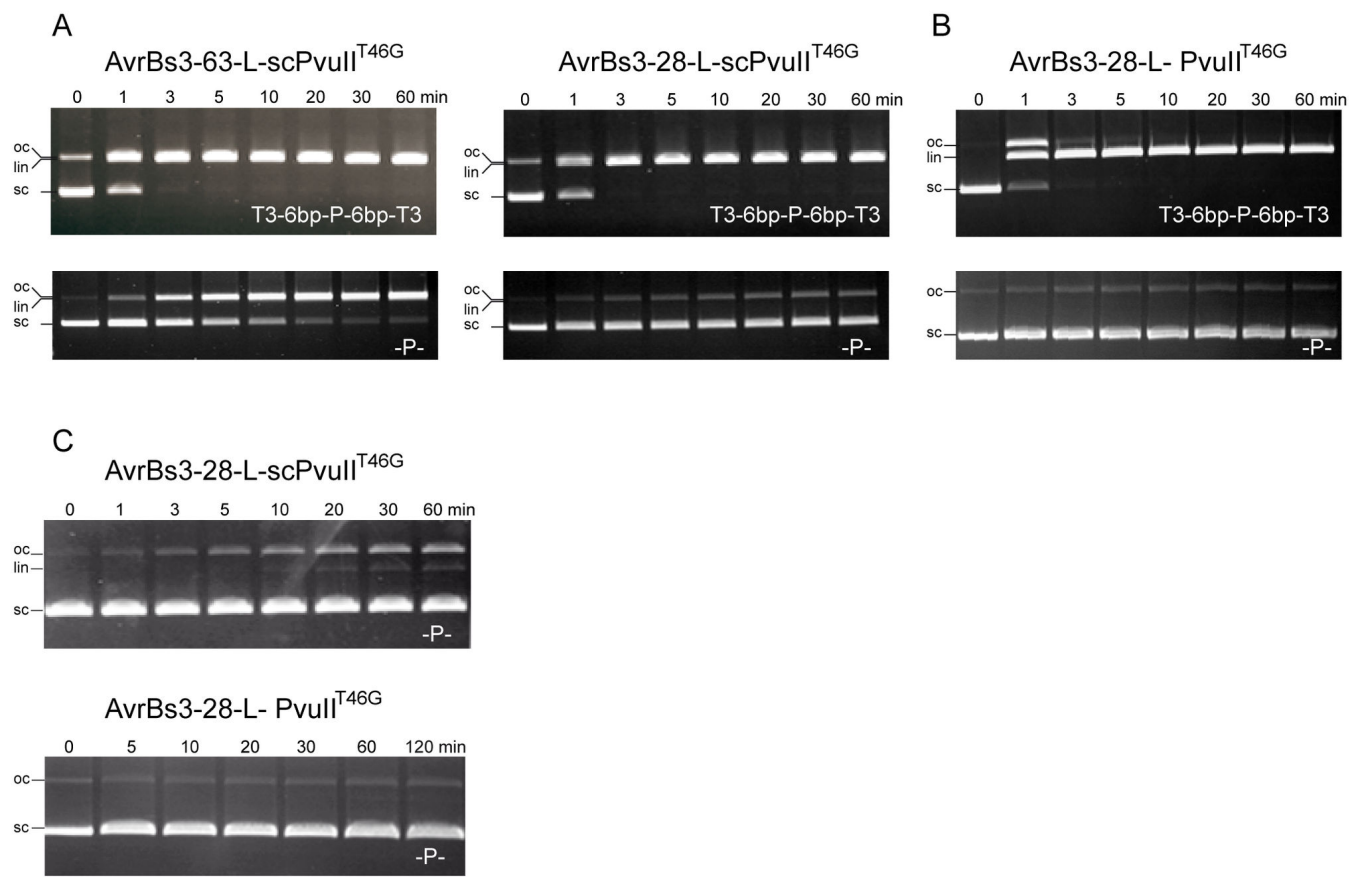
Analysis of the cleavage activity of AvrBs3-PvuII fusion proteins. (**A**) **and **(**B**) Comparison of the cleavage rates of selected AvrBs3-PvuII fusion proteins (as indicated) under low ionic strength: 76 mM (20 mM Tris-Ac, 50 mM K-Ac, 2 mM Mg-Ac, pH 7.5). In the top row the cleavage of the addressed substrate (T3-6bp-P-6bp-T3) is shown, in the bottom row that of the unaddressed substrate (-P-). All cleavage experiments were done with 8 nM DNA and 8 nM enzyme. (**C**) Comparison of the cleavage rates of an unaddressed substrate by selected AvrBs3-PvuII fusion proteins (as indicated) under physiological ionic strength: 143 mM (20 mM Tris-Ac, 120 mM K-Ac, 1 mM Mg-Ac, pH 7.5). The experiments were done with an excess of enzyme, the TALE-scPvuII fusion protein (top, 60 nM enzyme, 6 nM DNA) shows a higher cleavage activity with an unaddressed substrate (-P-) than the homodimeric TALE-PvuII^T46G^ fusion protein (bottom, 80 nM enzyme, 8 nM DNA). See the appearance of nicked and linearized DNA with AvrBs3-28-L-scPvuII^T46G^. There is no nicking or cleavage detectable of the unaddressed substrate with AvrBs3-28-L-PvuII^T46G^. oc, open circle; lin, linearized; sc, supercoiled.

As the length of the linker between the binding and cleavage modules plays an important role for activity and specificity of fusion proteins [53,54], we have compared two linkers, 63-L and 28-L, to span the distance between the C-terminus of AvrBs3 and the N-terminus of PvuII. Linker 63-L consists of the 63 amino acids following the last AvrBs3 DNA-binding repeat and the 16 residues long linker L (see Materials and Methods). In linker 28-L only 28 residues following the last repeat of AvrBs3 are present. The DNA cleavage activity of the fusion proteins was measured with a supercoiled plasmid with an addressed site (T3-6bp-P-6bp-T3) and for comparison with a supercoiled plasmid with an unaddressed PvuII site (-P-). Figure 2 shows that all fusion proteins tested (8 nM) cleave the supercoiled plasmid (8 nM) with an addressed site to create a linearized plasmid within minutes at low ionic strength (76 mM). Under the same conditions, the plasmid substrate with an unaddressed site is also cleaved, albeit significantly more slowly. It is noteworthy that the fusion protein with the short linker is more specific than the fusion protein with the long linker (Figure 2A). We have therefore used the fusion proteins with the short 28-L linker in all subsequent experiments. Furthermore, the PvuII fusion protein seems to be slightly more specific than the scPvuII fusion protein (Figure 2A and B).

### Ionic strength affects the specificity of TALE-PvuII fusion proteins

Higher ionic strength suppressed non-specific DNA cleavage by ZFN-PvuII fusion proteins [54]. Therefore we tested if TALE-PvuII fusion proteins would show higher specificity at ionic strength comparable to physiological conditions. We found that at an ionic strength of 143 mM, AvrBs3-28-L-PvuII^T46G^ did not cleave unaddressed PvuII sites in detectable amounts even at a 10-fold excess of enzyme (80 nM) over substrate (8 nM). The scPvuII^T46G^ variant was somewhat less specific as compared to the PvuII^T46G^ variant (Figure 2C). Thus, similar to ZFN-PvuII fusion proteins, TALE-PvuII fusions showed enhanced specificity at physiological ionic strength. All subsequent experiments were therefore carried out with AvrBs3-28-L-PvuII^T46G^ in a buffer with an ionic strength of about 150 mM.

### The presence of the PvuII site is essential for cleavage by TALE-PvuII fusion proteins

**Figure 3:**
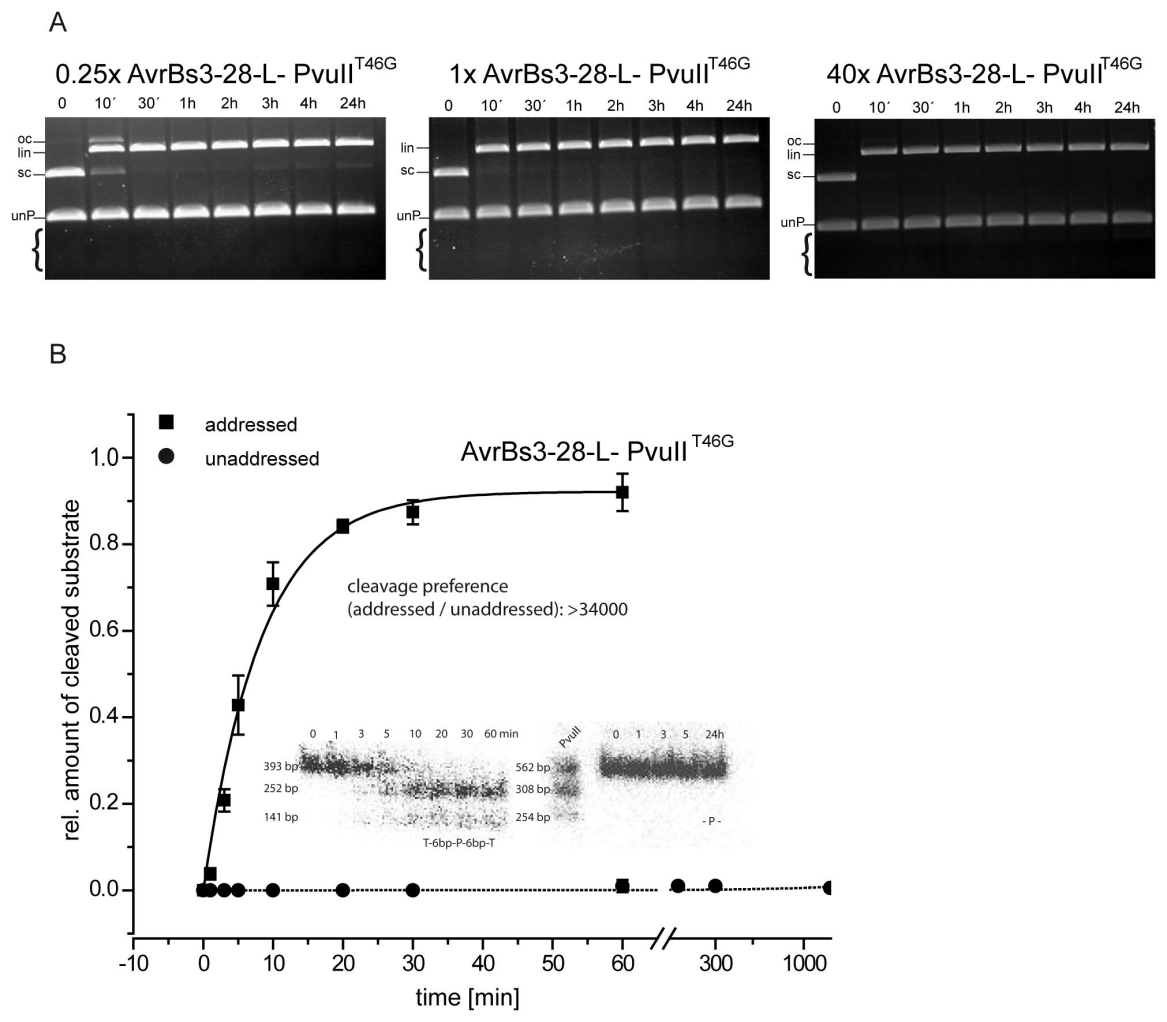
Analysis of competition cleavage experiments with AvrBs3-PvuII fusion proteins. (**A**) Competition cleavage experiments with AvrBs3-28-L-PvuII^T46G^ under physiological ionic strength. Shown is the cleavage pattern with supercoiled plasmid DNA with an addressed site (8 nM) in competition with a PCR fragment (unP) with an unaddressed site (32 nM). The experiment was carried out with a variable excess of enzyme over plasmid substrate (0.25 to 40-fold). The enzyme shows complete cleavage of the addressed substrate but no cleavage of the unaddressed substrate, even in an overnight incubation with a 40-fold excess of enzyme over the addressed plasmid substrate (8 nM) and 10-fold excess over the unaddressed PCR substrate (32 nM). The brackets indicate the positions where one would expect the products of cleavage of the unaddressed PCR substrate. oc, open circle; lin, linearized; sc, supercoiled. (**B**) Quantitative determination of the preference of AvrBs3-28-L-PvuII^T46G^ for an addressed (T3-6bp-P-6bp-T3) over an unaddressed site (-P-). The reactions were performed in triplicate under physiological conditions with 20 nM enzyme and 20 nM addressed substrate (squares) and unaddressed substrate (circles), both PCR fragments were radioactively labelled with [α^32^P]dATP. The insert shows the primary data: the electrophoretic analysis of the cleavage reaction products using an Instant Imager. From the fit, a cleavage preference of > 34,000-fold was determined.

For a quantitative determination of the specificity of AvrBs3-28-L-PvuII^T46G^, varying concentrations of enzyme were challenged with a mixture of a specific substrate (8 nM plasmid DNA with an addressed PvuII site) and a non-specific substrate (32 nM PCR fragment with an unaddressed PvuII site). As shown in Figure 3A, regardless of the relative concentrations of enzyme and substrate (from 0.25:1 to 40:1) only cleavage of the addressed site could be observed. Even at a 40-fold excess of enzyme over addressed sites (40:1) and long incubation times, cleavage of the unaddressed site was not detectable. It is noteworthy that even with an excess of substrate over enzyme, complete cleavage of substrate is observed, demonstrating that the TALE-PvuII fusion protein exhibits turnover.

For a quantitative evaluation of the preference of AvrBs3-28-L-PvuII^T46G^ for an addressed over an unaddressed site, radioactively labelled PCR fragments were used. Figure 3B shows the kinetics of cleavage of 20 nM PCR fragments with an addressed site and an unaddressed site, respectively, by 20 nM enzyme. Taking the limits of detection into consideration, the preference of AvrBs3-28-L-PvuII^T46G^ for an addressed over an unaddressed site was >34,000-fold (Figure 3B). This preference was even superior than the one we had previously measured for the inactive I-SceI and zinc finger versions of the PvuII fusions, namely >1,000-fold [53,54].

### Target site requirements for cleavage by TALE-PvuII fusion proteins

**Figure 4:**
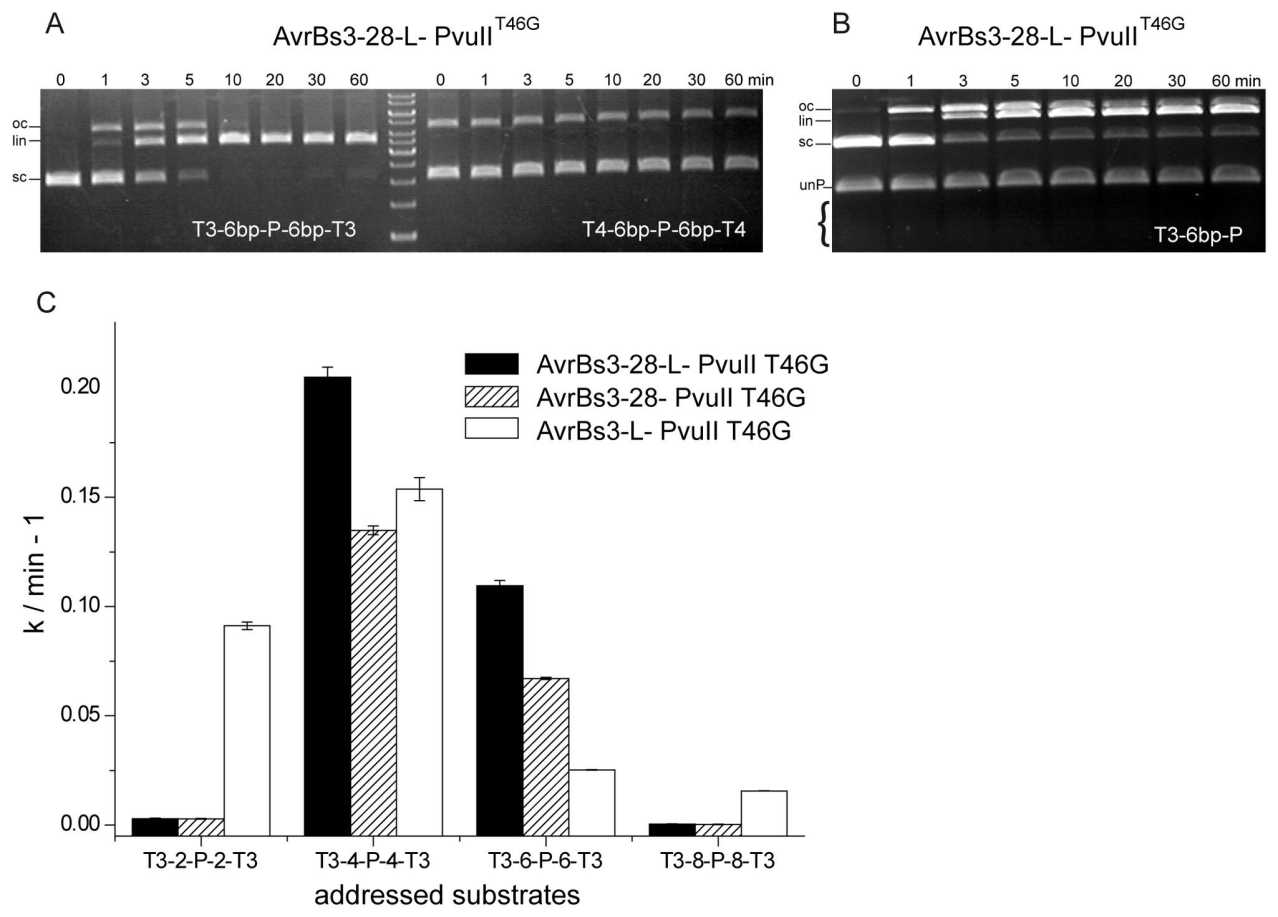
Analysis of the cleavage activity of AvrBs3-PvuII fusion proteins on AvrBs3 and AvrBs4 substrates. (**A**) Specificity of cleavage analyzed with the T3-6bp-P-6bp-T3 substrate and the T4-6bp-P-6bp-T4 substrate which differ in 11 (8, respectively, considering the degeneracy of the TALE recognition code) out of 19 positions from the AvrBs3 target site. No nicking or cleavage of the AvrBs4 substrate (8 nM) by AvrBs3-28-L-PvuII^T46G^ (8 nM) could be detected. (**B**) Cleavage of a “half-site” substrate by AvrBs3-28-L-PvuII^T46G^. The “half-site” substrate is a bipartite substrate consisting of an AvrBs3 recognition site and a PvuII recognition site (T3-6bp-P). The sc plasmid (8 nM) with the “half-site” was incubated with an equimolar concentration of AvrBs3-28-L-PvuII^T46G^ (8 nM). The assay was done under physiological ionic strength and in competition with a 32 nM PCR fragment (unP) with one unaddressed PvuII site (-P-). Whereas the “half-site” substrate is cleaved almost to completion, the unaddressed PCR fragment is not cleaved at all. (**C**) The effect of the distance of the AvrBs3 and the PvuII site on the rate of DNA cleavage by various AvrBs3-PvuII fusion proteins. 20 nM radioactively labelled PCR fragments with 2 (T3-2-P-2-T3), 4 (T3-4-P-4-T3), 6 (T3-6-P-6-T3) and 8 (T3-8-P-8-T3) bp between the AvrBs3 and the PvuII site were incubated with 20 nM AvrBs3-28-L-PvuII^T46G^, AvrBs3-28-PvuII^T46G^ and AvrBs3-L-PvuII^T46G^ for 60 min.

So far, only the specificity of AvrBs3-28-L-PvuII^T46G^ for a target sequence comprising the AvrBs3 and a PvuII site (T3-6bp-P-6bp-T3) was analyzed. We were interested to know whether AvrBs3-28-L-PvuII^T46G^ would also cleave a target sequence comprising an AvrBs4 recognition site. AvrBs4 has the same overall architecture as AvrBs3 but its DNA target sequence differs in 8 out of 19 positions from the AvrBs3 site. Figure 4A shows that at equimolar concentrations of enzyme and substrate (8 nM) only the AvrBs3 but not the AvrBs4 site-containing DNA target (T4-6bp-P-6bp-T4) was first nicked and then cleaved.

The experiments reported above were all carried out with a “tripartite” substrate that contains a central PvuII site and two flanking AvrBs3 target sites (T3-6bp-P-6bp-T3). We wondered, whether a “bipartite” substrate that lacks one of the two flanking AvrBs3 target sites (T3-6bp-P) would also be cleaved by AvrBs3-28-L-PvuII^T46G^. Figure 4B shows that the bipartite substrate (8 nM) at equimolar concentration with the enzyme was cleaved almost as efficiently as the tripartite substrate, whereas an unaddressed substrate was not cleaved (Figure 2B). A substrate lacking the central PvuII site is not cleaved (data not shown).

It cannot be precisely predicted by modeling of the TALE-PvuII^T46G^ fusion protein (Figure 1B), what the optimal distance between the AvrBs3 and PvuII site for double-strand cleavage by the AvrBs3-28-L-PvuII^T46G^ fusion protein would be. We have therefore produced several DNA substrates with 2, 4, 6 and 8 bp between the AvrBs3 and the PvuII site. As shown in Figure 4C, the distance of 4 and 6 bp is favoured over the others, not only for AvrBs3-28-L-PvuII^T46G^ (44-aa linker), but also for AvrBs3-28-PvuII^T46G^ (28-aa linker), which has a shorter linker between the AvrBs3 repeat array and PvuII module. AvrBs3-L-PvuII^T46G^ (16-aa linker) with the shortest linker, however, preferred the substrate with the 2-bp linker over the one with the 6-bp linker. The different linkers had no influence on the specificity of the fusion protein (data not shown).

### Activity and toxicity of TALE-PvuII fusion proteins in human cells

**Figure 5:**
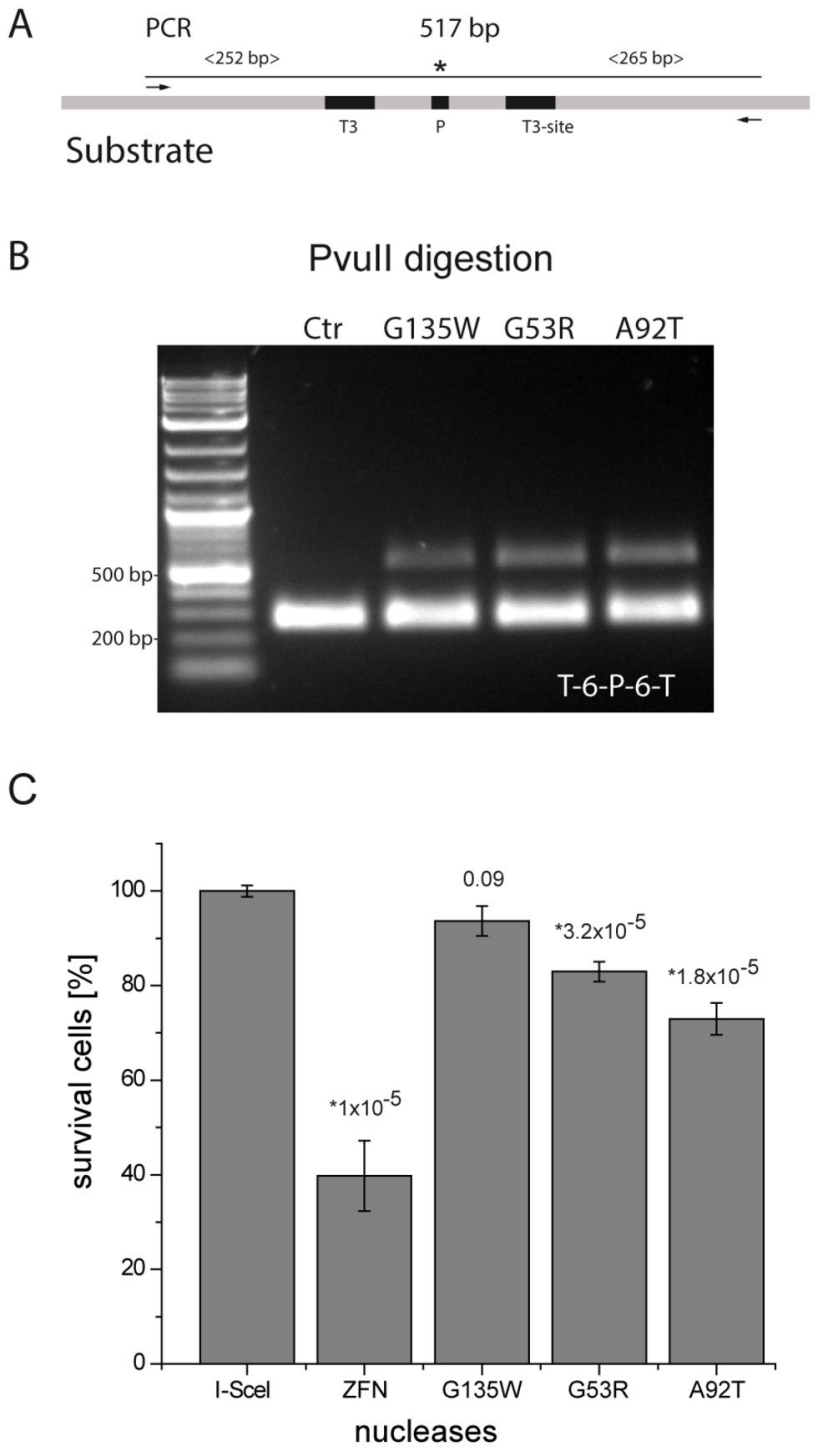
Activity and toxicity of TALE-PvuII fusion proteins in human cells. (**A**) PCR was performed with the plasmid from the HEK293 cells resulting in a DNA fragment of 517 bp. * indicates the cleavage site of PvuII. (**B**) Analysis of the PCR product (14.5 nM) after digestion with 20 U of PvuII for 1 h. A cleavage-resistant band indicates the loss of the PvuII site by NHEJ and confirms the activity of the TALE-PvuII fusion proteins. (**C**) Cell toxicity of the PvuII-based TALENs. After co-transfection of a mCherry expression plasmid, cell survival rate was calculated as the decrease in the number of mCherry-positive cells from day 2 to day 5 by flow cytometry, normalized to cells transfected with an I-SceI expression vector. * Statistically significant differences in toxicities between I-SceI and TALE-PvuII fusion proteins are indicated (P-values) .

We routinely check the activity of our PvuII fusion proteins in a viability assay in *E. coli* before testing their activity in human cells. In the viability assay, *E. coli* cells not expressing the PvuII DNA-methyltransferase (M.PvuII) are challenged with the PvuII fusion proteins. Clones growing in the absence of M.PvuII and in the presence of TALE-PvuII are picked. Some of them are subjected to further analysis: the sequence of the open reading frame coding for the TALE-PvuII protein is determined and the activity of the protein analyzed *in vitro*. The most active ones are then subjected to a cellular plasmid cleavage assay: in the present study G53R, A92T, G135W. As shown in Figure 5, the three AvrBs3-PvuII variants, which also carry the high fidelity mutation T46G, were active in HEK293 cells co-transfected with a plasmid carrying the target site for AvrBs3-PvuII and one of the respective nuclease expression vectors [54]. In human cells, target site (in this case T3-6bp-P-6bp-T3, Figure 5A) cleavage by the engineered nuclease is followed by error-prone NHEJ repair, leading to insertions and deletions at the target site, which can be detected by PCR followed by restriction with PvuII (Figure 5B). The partial resistance of the PCR fragment to cleavage with PvuII demonstrates that some of the isolated target plasmids had been cleaved by the TALE-PvuII variant. The activity assay showed that the three variants, AvrBs3-28-L-PvuII^T46G;G53R^, AvrBs3-28-L-PvuII^T46G;A92T^ and AvrBs3-28-L-PvuII^T46G;G135W^ had similar activity in human cells. Nuclease-associated cytotoxicity was determined by co-transfecting an mCherry expression plasmid and comparing the decrease of mCherry positive cells to a control nuclease [63]. Figure 5C shows that the three AvrBs3-PvuII variants induced little cytotoxicity which was slightly higher than that of I-SceI but considerably less than that of the ZFN pair GZF1N/GZF3N [64].

## Discussion

**Figure 6:**
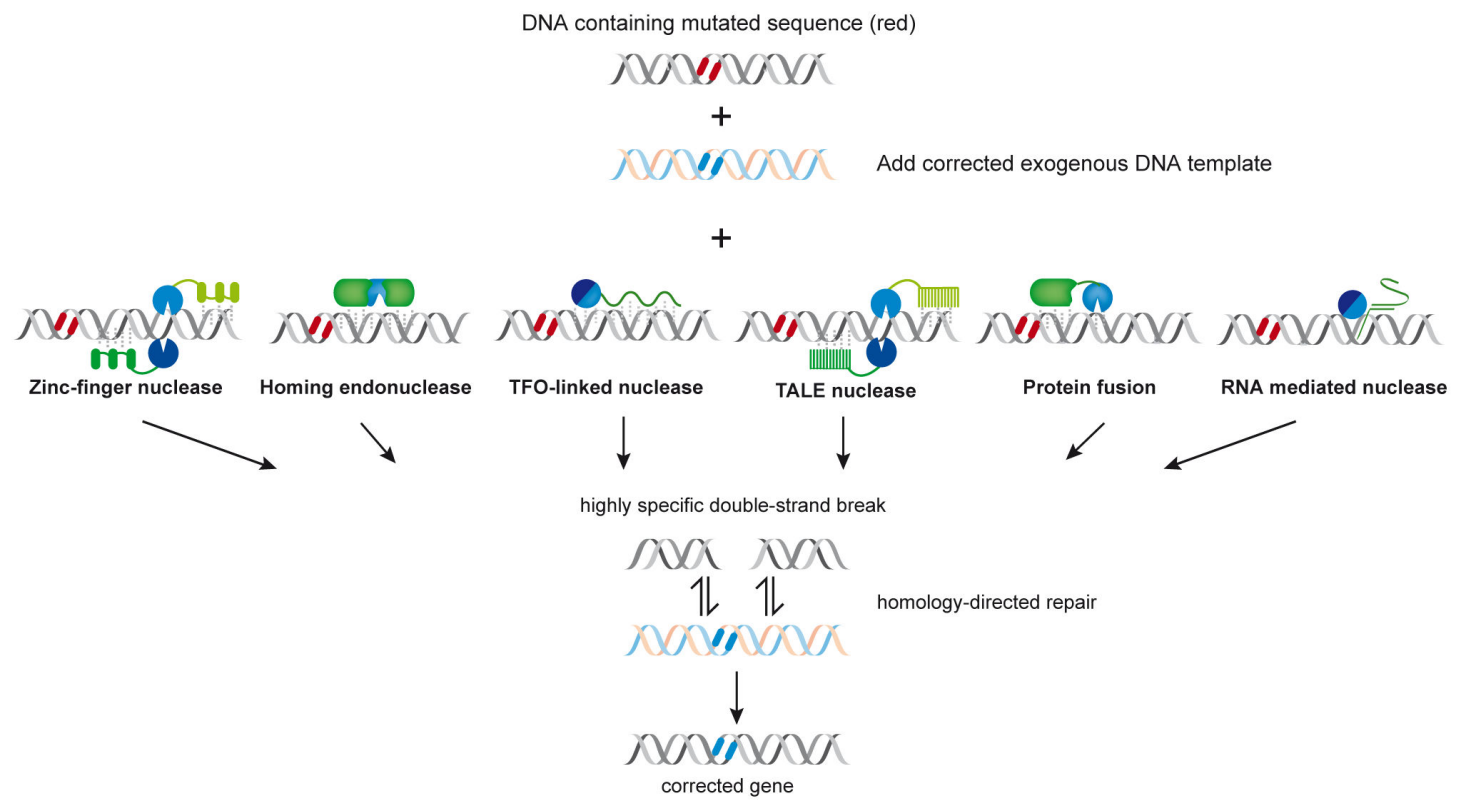
Engineered highly specific endonucleases that can be used for gene targeting by introducing a double-strand break into a complex genome and thereby stimulating homologous recombination. With the exception of engineered homing endonucleases (“meganucleases”) in which the function of DNA binding and DNA cleavage is present in the same polypeptide chain [77], the other engineered nucleases consist of separate DNA-binding (green) and DNA-cleavage (blue) modules. Zinc finger nucleases and TALE nucleases usually have the non-specific cleavage domain of the restriction endonuclease FokI as DNA-cleavage module, but as shown recently and in the present paper the restriction endonuclease PvuII can also be used for this purpose [54]. PvuII has also been employed in TFO-linked nucleases [49] and in protein fusions (with catalytically inactive I-SceI) [53] as DNA-cleavage module. Zinc finger nucleases, TALE nucleases and TFO-linked nucleases are programmable, as are the RNA-mediated nucleases [36] [modified after [3]] .

Genome editing with engineered nucleases was chosen as Method of the Year 2011 by the editors of Nature Methods, as it is a powerful tool for studying biological processes [67]. In principle, it has also the potential to be used for gene targeting in general and gene therapy in particular, for example to correct mutations in monogenic hereditary human diseases by targeted approaches based on homologous recombination [7,68]. A variety of engineered nucleases are being used or considered for this purpose (Figure 6). Here, we introduce a new architecture for engineered nucleases to be used for genome editing.

The most widely used programmable nucleases are ZFNs and TALENs followed by the recently introduced CRISPR/Cas-based nucleases [4]. ZFNs and TALENs differ in their DNA-binding module but have the same DNA-cleavage module, the non-specific catalytic domain of the Type IIS restriction endonuclease FokI. The natural FokI enzyme is a monomeric protein, both free in solution and when bound to DNA in the absence of divalent metal ions [43,69]. A flexible linker connects the DNA-recognition domain with the non-specific DNA-cleavage domain. For DNA cleavage, FokI dimerization, which occurs via the catalytic domain, is required [42]. A productive complex can be formed by various ways [70]: (i.) two FokI monomers free in solution can associate to form a dimer which then binds to its recognition sequence GGATG and, in the presence of Mg^2+^ ions, cleaves the DNA downstream of the recognition site, 9 and 13 nucleotides away in the top and bottom strand, respectively. (ii.) FokI binds to its recognition site as a monomer and recruits another FokI monomer free in solution forming a dimer which cleaves the DNA in the presence of Mg^2+^ ions. (iii.) On a DNA substrate with two (or more) recognition sites, the active dimer can be formed from two FokI monomers each bound to a recognition site, which means that the DNA between the two recognition sites is looped out [71,72]; again, cleavage occurs only in the presence of Mg^2+^ and is considerably faster on a substrate with two (or more) recognition sites than on a substrate with one recognition site [72]. The various ways by which FokI can form the active dimer implies that cleavage of a ZFN target, i.e. two ZF recognition sites located adjacent to each other, can occur, though rarely, by cleavage at half-sites, i.e. at only one ZF recognition site [44]. This would cause so called off-target cleavage resulting in unacceptable toxicity. In contrast to classical FokI-based ZFN and TALENs, PvuII-based TALENs are dimeric proteins such as the natural PvuII, both in the absence [73] and the presence of a substrate [74], and thus do not require dimerization on the target, which eliminates one source for off-target cleavage. Off-target cleavage can be greatly reduced, but not fully abolished by rationally redesigning the ZFN dimer interface to inhibit homodimerization [45,46,75]. Off-target cleavage specificities of optimized ZFNs were analyzed by Gabriel et al. [47] and Pattanayak et al. [48]. *In vivo* cleavage sites were identified that resembled the intended target site but could not be predicted *in silico*. It must be emphasized that off-target cleavage is likely to also be a problem with other engineered nucleases that use the catalytic domain of FokI as DNA-cleavage module, such as TALENs.

After we had recently shown that in ZFNs the catalytic domain of FokI can be replaced by PvuII [54], we now demonstrate that also in TALENs PvuII can take over the role of the DNA-cleavage module. Substituting the non-specific domain of FokI for PvuII (or other specific nucleases) has the advantage that an extra element of specificity, the PvuII recognition site CAGCTG, is added to the TALE protein. The challenge of designing TALE-PvuII fusion proteins as highly specific programmable nucleases is to take precautions that a PvuII site that is not addressed by a TALE protein recognition site is not attacked by the PvuII module in the TALE-PvuII fusion protein. There are in principle two different strategies to achieve this, as shown for ZF-PvuII [54] and I-SceI*-PvuII [53] fusion proteins: (i.) One could weaken the interaction of PvuII to the PvuII recognition site by introducing amino acid substitutions, such that PvuII as the cleavage module in the fusion protein is absolutely dependent on the TALE protein for a productive interaction with the composite site consisting of a TALE recognition site and a PvuII recognition site, i.e. the addressed PvuII recognition site. (ii.) Another possibility is to construct the linker between the DNA-binding and DNA-cleavage module such that PvuII cannot make a productive interaction with a PvuII site, unless the TALE protein has made contact with its binding site and thereby released the DNA cleavage module PvuII from an inhibited conformation. We believe that in the TALE-PvuII construct that we have produced, PvuII presumably is sterically hindered by the TALE protein to form an activated transition state complex with the PvuII recognition site, unless there is a strong interaction between the DNA binding domain of the TALE protein and the TALE target site.

PvuII is a homodimeric protein; this means that also the TALE-PvuII fusion proteins are homodimers. Because a single chain version (sc) of PvuII has been described [59], we also generated monomeric TALE-scPvuII fusion proteins. We have prepared several versions of the TALE-PvuII fusion protein, all of which carry the T46G mutation which exhibits less star activity than wildtype PvuII [58]. The TALE-PvuII fusion proteins were tested on tripartite substrates which contained a PvuII site between two TALE recognition sites, e.g. TALE-6 bp-PvuII-6 bp-TALE. The homodimeric TALE-PvuII with the 28-L linker turned out to be more specific, i.e. showed less unaddressed cleavage, than the single chain variant and the variant with the 63-L linker. Therefore, all subsequent experiments were done with the homodimeric fusion protein with the short linker. At physiological ionic strength (~150 mM), no unaddressed cleavage was observed, not even at excess of enzyme (up to 10-fold) over substrate and long incubation times (up to 24 hours in a plasmid cleavage assay). The specificity of the TALE-PvuII fusion protein was quantified with radioactively labelled addressed and unaddressed substrates and found to be >34,000-fold for the addressed substrate. The TALE-PvuII fusion protein prefers a spacer length (distance between TALE and PvuII recognition sequences) of 4 to 6 bp. 

Remarkably, the TALE-PvuII fusion protein also accepts half-sites, i.e. bipartite recognition sites, e.g. TALE-6bp-PvuII. We suggest that binding of a single AvrBs3 module to the TALE binding site of the substrate, leaving the other AvrBs3 module making non-specific contacts with the DNA, is sufficient to activate PvuII. This may be due to the rather high ratio of enzyme vs. substrate *in vitro* which can hardly be achieved *in cellula*. Still, this finding implies that precision genome engineering can be done by cleavage of a site consisting of one TALE target site adjacent to a PvuII site. If they are sufficiently specific, this would be an advantage over TALENs (and ZFNs) based on the catalytic domain of FokI, which require heterodimerization of two proteins with different target recognition domains. However, we have recently also engineered active obligate heterodimeric variants of TALEN-PvuII (Yanik et al, in preparation). This will allow us to target extended recognition sequences.

The TALE-PvuII fusion proteins that we have produced are highly active and specific *in vitro*. To answer the question whether they are active *in vivo*, we have tested them in human cells using a cellular plasmid cleavage assay. This assay showed that all TALE-PvuII fusion proteins were active in HEK293T cells. When assaying cytotoxicity associated with expression of the TALE-PvuII fusion proteins, they exhibited almost as little toxicity as I-SceI and performed better than a toxic reference ZFN. PvuII-based TALENs (and ZFNs) require the presence of a PvuII site as part of a potential target site, which may be considered a disadvantage. However, since PvuII sites statistically occur on average within a few thousand base pairs, it should be possible to find a suitable target sites for TALE-PvuII fusion proteins.

## Conclusions

In the present paper, we demonstrated that TALENs can be produced with the restriction endonuclease PvuII as DNA-cleavage module instead of the most frequently used non-specific cleavage domain of FokI. The TALE-PvuII fusion protein does not exhibit any non-specific cleavage at unaddressed PvuII sites nor at any other site, as was demonstrated in experiments in which DNA carrying unaddressed PvuII sites was incubated with a high molar excess of the TALE-PvuII fusion protein for up to 24 hours. Under these conditions, addressed PvuII sites were cleaved within minutes. In principle, TALE-PvuII fusion proteins can be produced as homodimers or as monomers with scPvuII. The TALE-PvuII fusion proteins turned out to be active in HEK293T cells, with almost as little toxicity as I-SceI. Taken together, we suggest that TALE-PvuII fusion proteins should be considered as suitable alternatives to classical ZFNs and TALENs. Of course, highly specific nucleases, regardless of which DNA-cleavage module they contain, need to be tested *in vivo* for their specificity which means for off-target cleavage, as it was done for ZFNs in a genome-wide analysis [47,48]. Finally, we have now expanded the “tool box” for gene targeting by a double-strand specific TALE-PvuII nuclease, which might be the nuclease of choice for gene disruption, whereas a nicking nuclease, such as TALE-MutH [57], might be more useful for gene replacement where one wants to avoid non-homologous end joining (NHEJ). 

## Supporting Information

Figure S1 **Analysis of the final protein purification step by SDS-polyacrylamide gel electrophoresis.**
(PDF)

Table S1 **Overview of the substrates and primers used.**
(PDF)
