# Supplementary material for: TALE-PvuII Fusion Proteins – Novel Tools for Gene Targeting
Source: PLoS One. 2013 Dec 5;8(12):e82539. doi: 10.1371/journal.pone.0082539 (PMC3857828; doi:10.1371/journal.pone.0082539)
Supplement: Figure S1 — Analysis of the final protein purification step by SDS-polyacrylamide gel electrophoresis. (PDF) [file pone.0082539.s001.pdf]

Supplementary Figure S1

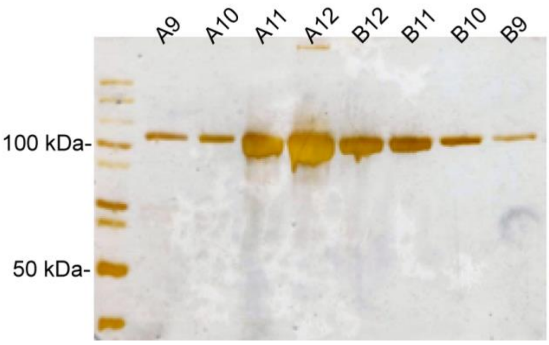

Figure S1: Analysis of the final purification step by SDS-polyacrylamide gel electrophoresis and silver staining. The gel shows the TALE-scPvuII fractions after ion exchange chromatography with a heparin column. Fractions A11-B11 were pooled for further analysis.
