# Supplementary material for: TALE-PvuII Fusion Proteins – Novel Tools for Gene Targeting
Source: PLoS One. 2013 Dec 5;8(12):e82539. doi: 10.1371/journal.pone.0082539 (PMC3857828; doi:10.1371/journal.pone.0082539)
Supplement: Table S1 — Overview of the substrates and primers used. (PDF) [file pone.0082539.s002.pdf]

## *Table S1: Overview of the substrates and primers used*

| ***Plasmid substrates*** | ***target sequences*** |
|---|---|
| T3-2-P-2-T3* | 5‘ TTTATCTATAAACCTAACCCTCTTG**CAGCTG**CGAGAGGGTTAGGTTTATAGATAAACATG 3‘ |
| T3-4-P-4-T3 | 5‘ TTTATCTATAAACCTAACCCTCTCCTG**CAGCTG**CGGGAGAGGGTTAGGTTTATAGATAAACATG 3 |
| T3-6-P-6-T3 | 5‘ TTTATCTATAAACCTAACCCTCTTTCCTG**CAGCTG**CGGGAAAGAGGGTTAGGTTTATAGATAAACATG 3‘ |
| T3-8-P-8-T3 | 5‘ TTTATCTATAAACCTAACCCTCTTATTCCTG**CAGCTG**CGGGAATAAGAGGGTTAGGTTTATAGATAAACATG 3‘ |
| T3-6-P | 5‘ TTTATCTATAAACCTAACCCTCTGGATCC**CAGCTG**TCTAGACATG 3‘ |
| T4-6-P-6-T4 | 5’ TATAATTAATAATCCACTTGGATCC**CAGCTG**TCTAGAAAGTGGATTATTAATTATACATG 3’ |
| -P- | 5’ **CAGCTG** 3´ |
| T3-18-T3 | 5’ TCTATAAACCTAACCCTCTGGATCCGATATCTCTAGAAGAGGGTTAGGTTTATAGACATG 3’ |
| | |
| ***PCR substrates*** | ***primer pairs used*** |
| T3-n-P-n-T3 / T3-6-P (~390 bp) | 5´ CCGCCCAGTCCTGCTCGCTTC 3´ / 5´ ACCCAGAGCGCTGCCGGCAC 3´ |
| -P- 562 bp | 5´ GTATCACGAGGCCCTTTCGTCT 3´ / 5´ TTAGGAAGCAGCCCAGTAGTAG 3´ |
| -P- 900 bp | 5´ AAAACAGGAAGGCAAAATGC 3´ / 5´ TTAGGAAGCAGCCCAGTAGTAG 3´ |

*T3 is the wt AvrBs3 recognition site which was substituted in positions 2 and 16 by exchanging A →C, and 19 C →T following the RVD preference as indicated by Boch et al. [21]
